# Next‐generation proteomics improves lung cancer risk prediction

**DOI:** 10.1002/1878-0261.70166

**Published:** 2025-11-20

**Authors:** Megha Bhardwaj, Clara Frick, Ben Schöttker, Bernd Holleczek, Hermann Brenner

**Affiliations:** ^1^ Clinical Epidemiology of Early Cancer Detection German Cancer Research Center (DKFZ) Heidelberg Germany; ^2^ Heidelberg Medical Faculty Heidelberg University Heidelberg Germany; ^3^ Saarland Cancer Registry Saarbrücken Germany; ^4^ Cancer Prevention Graduate School German Cancer Research Center (DKFZ) Heidelberg Germany

**Keywords:** lung cancer, population‐based cohort, proteins, risk model, risk prediction, screening

## Abstract

Screening heavy smokers by low‐dose computed tomography (LDCT) can reduce lung cancer (LC) mortality, but defining the population that benefits most, a prerequisite for cost‐effective screening, is challenging. In order to contribute to a more nuanced risk stratification of high‐risk target populations, we developed and validated a blood‐based protein marker model for LC. A two‐stage design was implemented in this study, and the derivation set comprised 18 868 participants from the UK Biobank, which included 200 incident LC cases identified at 6 years of follow‐up. The independent validation set included 101 LC cases identified at 6 years of follow‐up. A total of 2025 protein markers measured by proximity extension assays available for both datasets were used for analysis. A risk prediction algorithm by least absolute shrinkage and selection operator regression with bootstrap method was developed in the derivation set and then externally evaluated in the independent validation set. The risk discriminatory performance of the protein marker model was compared with the established PLCO_m2012_ model, USPSTF 2020 guidelines and trial criteria used in different LDCT trials. The protein marker model comprising of four protein biomarkers—CEACAM5, CXCL17, MMP12, and WFDC2—outperformed the PLCO_m2012_ model, and the areas under the receiver operating curve (AUCs) for the protein marker model in the derivation and validation sets were 0.814 [95% confidence interval (95% CI), 0.785–0.843] and 0.814 (95% CI, 0.756–0.873), respectively. The addition of the protein marker model to the PLCO_m2012_ model increased the AUCs up to 0.056 and 0.057 and yielded up to 16 and 12 percentage points higher sensitivities to identify future LC cases compared to the LDCT trial criteria, in the derivation and validation sets, respectively. The protein marker model improves the selection of high LC risk individuals for LDCT screening and thereby enhances screening efficacy.

## Graphical abstract

This is one of very few studies that used prediagnostic blood samples from participants of two large population‐based cohorts. We identified, evaluated, and validated an innovative protein marker model that outperformed an established risk prediction model and criteria employed by low‐dose computed tomography in lung cancer screening trials. These proteins demonstrate promising potential in identifying lung cancer cases up to 6 years before diagnosis.

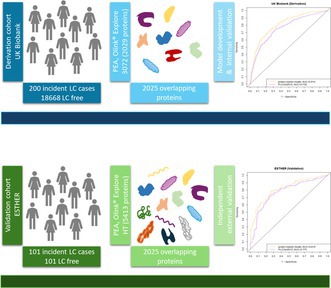

**Abbreviations**
AUC: area under the receiver operating curve
CEACAM5: carcinoembryonic antigen‐related cell adhesion molecule 5
CXCL17: C‐X‐C motif chemokine 17
DANTE: Detection and Screening of Early Lung cancer with Novel Imaging Technology
DLCST: Danish Lung Cancer Screening Trial
ESTHER: Epidemiologische Studie zu Chancen der Verhütung, Früherkennung und optimierten Therapie chronischer Erkrankungen in der älteren Bevölkerung
ITALUNG: Italian Lung Cancer Computed Tomography Screening Trial
LASSO: least absolute shrinkage and selection operator
LC: lung cancer
LDCT: low‐dose computed tomography
LUSI: German Lung Cancer Screening Intervention trial
MILD: Multicentric Italian Lung Detection Trial
MMP12: macrophage metalloelastase
NELSON: Nederlands‐Leuvens Longkanker Screenings Onderzoek trial
NLST: United States National Lung Screening Trial
NPX: normalized protein expression
PLCO_m2012_
: Prostate, Lung, Colorectal, and Ovarian Cancer Screening Trial Model 2012
STARD: Standards for the Reporting of Diagnostic Accuracy Studies
USPSTF: US Preventive Services Task Force
WFDC2: WAP four‐disulfide core domain protein 2

## 1 Introduction

Lung cancer (LC) is the leading cause of cancer mortality and the most common cancer globally, accounting for more than 1.8 million deaths and over 2.4 million cancer cases in 2022 [1]. Randomized control trials have demonstrated the effectiveness of screening high‐risk individuals with low‐dose computed tomography (LDCT) in reducing lung cancer mortality [2, 3, 4, 5, 6]. Various risk prediction models have been proposed, evaluated, and validated that when compared with previously suggested eligibility criteria might identify future LC cases and deaths with greater efficiency [7, 8, 9]. The PLCO_m2012_ model that incorporates a comprehensive set of variables, such as demographic information, smoking history, and medical history, has demonstrated strong performance in predicting LC risk across diverse populations, including American and European cohorts [10, 11, 12].

Blood‐based biomarkers that improve risk assessment may be useful as a prescreening eligibility test. Numerous biomarkers like proteins, microRNAs, and methylation of circulating tumor DNA have been proposed to enhance the selection of individuals for LC screening [13, 14, 15, 16]. A risk‐informative, biomarker‐based prescreening test could potentially identify individuals who are at high LC risk despite not meeting current eligibility criteria. The current study aimed to evaluate if a blood‐based protein marker risk prediction model can improve the LC risk discriminatory performance of the established risk prediction model. For the derivation and independent external validation, the participants were selected from population‐based cohorts from the UK and Germany, using data and samples from the UK Biobank and the ESTHER cohort study, respectively. The proteins were quantified via proximity extension assay, a multiplex immunoassay in which paired oligonucleotide‐tagged antibodies binding the same antigen bring their DNA tags into proximity, allowing enzymatic extension and amplification for sensitive and specific protein measurement [17].

## 2 Materials and methods

### 2.1 Study design

The protein marker model was developed in a two‐step approach, with selection of proteins and construction of a multi‐marker algorithm in a derivation set and independent evaluation and validation of findings in an external validation set. The derivation set included participants from the UK Biobank study. The validation set was exclusively based on participants of the population‐based ESTHER cohort study.

### 2.2 Study population: derivation set

#### 2.2.1 The UK Biobank cohort

**Fig. 1:**
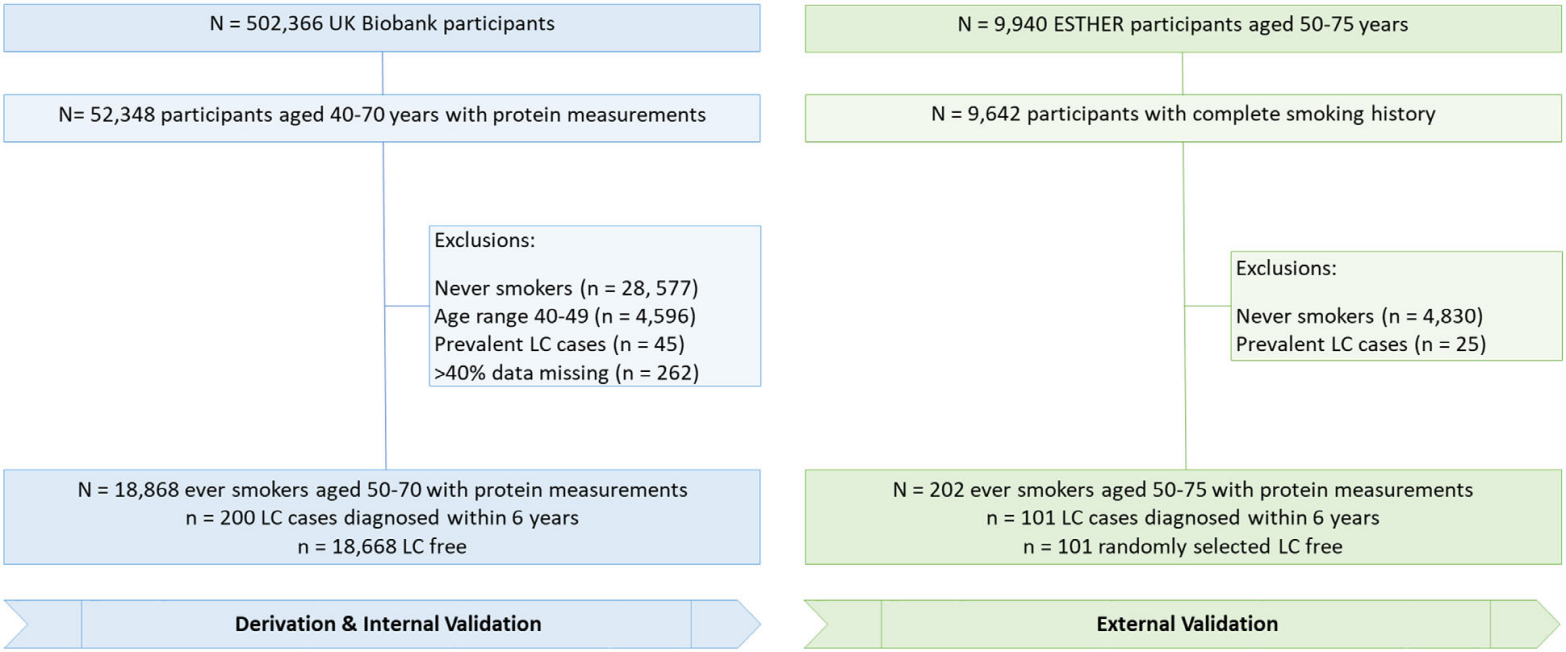
Flow diagram showing selection of study participants from UK Biobank and ESTHER. LC, lung cancer; *N*/*n*, number.

The UK Biobank study is a population‐based cohort study with over half a million adults recruited at ages 37–73 years across 22 assessment centers in England, Scotland, and Wales. All participants provided written informed consent and ethical approval was obtained from the North West‐Haydock Multicentre Research Ethics Committee (11/NW/0382), the National Information Governance Board for Health and Social Care in England and Wales, and the Community Health Index Advisory Group in Scotland. Detailed study protocols are available on the UK Biobank website (https://www.ukbiobank.ac.uk/). In brief, during the baseline recruitment visit between 2006 and 2010, biological samples (blood, stool, and urine) were collected in addition to information on sociodemographic, health and medical history, and anthropometric and lifestyle factors. Follow‐up of health‐related outcomes, including death and cancer, was conducted through linkage to electronic health records from the UK National Health Service. For the current study, ever‐smoking participants aged 50–70 with no prior LC diagnosis were selected from 52 348 participants included in the Pharma Proteomics Project for whom comprehensive proteomic profiles are available [18]. The complete Standards for the Reporting of Diagnostic Accuracy Studies (STARD) diagrams displaying the selection of study participants are shown in Fig. 1. The study population of the derivation set comprised of 200 ever‐smoking participants who were diagnosed with LC and 18 668 ever‐smoking participants without LC diagnosis during the 6 years of follow‐up.

### 2.3 Study population: validation set

#### 2.3.1 ESTHER

The participants in the validation set were selected from the ESTHER study (Epidemiologische Studie zu Chancen der Verhütung, Früherkennung und optimierten Therapie chronischer Erkrankungen in der älteren Bevölkerung) study, which is an ongoing state‐wide cohort study conducted in Saarland, Germany. Details of the ESTHER study design have been reported previously [19, 20]. Briefly, in the context of a general health screening examination, 9940 men and women aged 50–75 years were recruited by general practitioners in Saarland, Germany, between July 2000 and December 2002 and were regularly followed up thereafter. At baseline, information on sociodemographic characteristics, lifestyle factors, and health status was obtained by standardized self‐administered questionnaires from both participants and their general practitioners. Additionally, blood samples were collected and stored at −80 °C until analysis. The prevalence and incidence of all types of cancer cases at baseline and follow‐ups were determined by record linkage with data from the Saarland Cancer Registry (https://krebsregister.saarland.de/). All participants provided written informed consent, and the ESTHER study was approved by the ethics committees of the University of Heidelberg (Heidelberg, Baden‐Wuerttemberg, Germany) and of the state medical board of Saarland (Saarbruecken, Saarland Germany). The ESTHER study was conducted in adherence to the Code of Ethics of the World Medical Association (Declaration of Helsinki) for experiments involving humans. The evaluation of the protein markers in the current study was based on 101 ever‐smoking participants who were diagnosed with LC in the first 6 years of follow‐up and randomly selected 101 ever‐smoking participants without LC diagnosis during this time (Fig. 1).

### 2.4 Sample selection and storage

In the UK Biobank, blood was collected via venipuncture and the samples were centrifuged within 2 h of collection, aliquoted, and stored at −80 °C. In ESTHER, the blood samples were collected during the baseline survey, were transported to the laboratory within 24 h of collection, and then aliquoted and frozen at −80 °C. In both cohorts, there were no more than three freeze–thaw cycles until the protein measurements.

### 2.5 Proteomic assays and data processing

Protein profiling in blood samples in both the derivation and validation sets was performed utilizing antibody‐based proximity extension assays. Details about protein profiling in the UK Biobank have been published elsewhere [18]. Briefly, a total of 2 923 unique proteins were measured using Olink® Explore 3072 across eight protein panels. In the validation set comprising participants of ESTHER, the proteomic profiling of 5413 proteins was performed utilizing the Olink® Explore HT assays (which include almost all of the proteins covered by Olink® Explore 3072). Proximity extension assay has a broad dynamic range, being able to detect both high and low‐abundance proteins within the same assay and as the assay requires two antibodies binding to proximate epitopes and DNA extension, chances of nonspecific binding giving signal are reduced. Samples were processed across different plates, and each plate included a mix of study samples and controls to facilitate normalization and quality control [17].

For proximity extension assays performed in both the derivation and validation sets, no batch effects, plate effects, or abnormalities in protein coefficients of variation were observed. The complete preprocessing and selection of biomarkers is presented in Fig. S1. For data preprocessing, proteins with more than 25% of values below the limit of detection, with more than 20% missing data and those that were highly skewed were excluded. For further analysis, 2025 proteins that were overlapping and available in both the datasets were considered.

### 2.6 Heavy smoking definitions and prediction models

The criteria of heavy smoking used in previous LC screening trials and the US Preventive Services Task Force (USPSTF) guidelines like ≥ 30 pack‐years [2], ≥ 20 pack‐years [3, 21, 22, 23, 24], ≥ 15 cigarettes per day for ≥ 25 years or ≥ 10 cigarettes per day for ≥ 30 years [5, 25], and ≥ 15 cigarettes per day for ≥ 20 years [26] were used for comparison or combination of risk stratification in the current study. We furthermore used the Prostate, Lung, Colorectal, and Ovarian Cancer Screening Trial Model 2012 (PLCO_m2012_ model) [10].

### 2.7 Statistical analysis

The linear protein values were log transformed to produce normalized protein expression (NPX) and one NPX represents a two‐fold change in protein concentration. The NPX values of each individual protein were compared between LC cases and control participants without LC diagnosis during follow‐up using the Wilcoxon rank‐sum test with adjustment for multiple testing by the Benjamini and Hochberg method. A logistic regression model was used to construct the prediction algorithm for each protein and the prediction accuracy was evaluated by calculating areas under the receiver operating curve (AUCs) and their 95% confidence intervals (95% CI).

We used a predefined, multistep process combining univariate screening, penalized selection, and internal validation with bootstrapping to derive a protein marker model for prediction of incidence of LC. In the derivation set comprising UK Biobank participants, we first estimated the effective number of independent tests from a principal component analysis of the standardized NPX protein data using the Gao method and the significance threshold was adjusted by dividing 0.05 by effective number of tests. Only the protein biomarkers below this adjusted threshold in univariate logistic regression were carried forward. On the reduced set, we standardized predictors and applied least absolute shrinkage and selection operator (LASSO) with 10‐fold cross‐validation to select the tuning parameter. To enhance selection stability and correct for overfitting, we performed 1000 subsamples drawing 75% of observations each time, refitting LASSO, and recording variable inclusion and the protein biomarkers that were selected in at least 80% of replicates comprised the final set. We then carried out 1000 bootstrap splits (75% training, 25% testing) fitting logistic regression models on the final protein biomarkers and evaluating discrimination via area under the receiver operating characteristic curve (AUC), reporting the mean bootstrap adjusted (AUC*s). Finally, we fit the model on the full data using the selected markers to report its apparent performance AUCs not adjusted for overfitting and 95% CI. All random seeds were fixed for reproducibility. Furthermore, we assessed if and to what extent the combination of the protein marker model with PLCO_m2012_ could enhance LC prediction as compared to the risk model alone. The DeLong test was performed to assess whether the difference between the AUCs obtained for the PLCO_m2012_ model alone and for the combination of the protein marker model with the PLCO_m2012_ model was statistically significant.

The prediction potential of the protein marker model and the PLCO_m2012_ model, individually and in combination, to identify future LC cases, was compared with those of four different definitions of heavy smoking that were used as selection criteria by the LDCT trials and also the USPSTF 2020 guidelines. For that purpose, the cutoff of the models was adjusted to yield the same number of participants selected for LDCT screening as the respective trial criterion. Then, for each model, the sensitivity at this cutoff, defined as the proportion of incident LC cases selected for LDCT screening, was calculated and compared with the sensitivity of the respective trial criterion. McNemar test was performed to assess the statistical significance of the increase in sensitivity, as compared to the respective LDCT trial criterion. The comparative evaluations were further performed in an independent validation set consisting of ESTHER participants.

For the variables that had unknown information for some participants (for variables like years smoked, years quit, cigarettes per day, pack‐years of smoking, education, family history of lung cancer, lung diseases), multiple‐imputation datasets were created to impute these variables with the r package ‘mice’ [27]. All statistical analyses were performed with statistical software r language and environment (version 3.6.3, R Core Team) [28] using r packages ‘dplyr’, ‘DTComPair’, ‘glmnet’, ‘lcmodels’, ‘ModelGood’, ‘poolr’, and ‘pROC’. Statistical testing was two‐sided, and *P*‐values of 0.05 or less were considered to be statistically significant.

## 3 Results

The derivation set consisted of 200 incident LC cases and 18 668 ever‐smoking participants without LC diagnosis from UK Biobank participants. The validation set included 101 participants with LC diagnosis and 101 controls free of LC from the ESTHER study. Characteristics of the study populations from both the derivation and the validation sets are shown in Table 1. Incident LC cases were often older as compared to those free of LC in both derivation (median age: 65.0 vs. 61.0 years) and validation sets (median age: 65.0 vs. 60.0 years). Current smokers represented 49.0% and 54.5% of LC cases in derivation and validation sets, respectively.

**Table 1:** Characteristics of ever‐smoking participants. LC, lung cancer; *n*, number; SD, standard deviation.

Characteristics	UK Biobank		ESTHER	
	LC	LC free	LC	LC free
	*n* (%)	*n* (%)	*n* (%)	*n* (%)
	200	18 668	101	101
Age (years)				
50–59	40 (20.0)	7126 (38.2)	17 (16.8)	42 (41.6)
60–69	160 (80.0)	11 371 (60.9)	57 (56.4)	45 (44.6)
70–75	0 (0.0)	171 (0.9)	27 (26.7)	14 (13.9)
Mean (SD)	63.4 (4.6)	60.8 (5.4)	64.9 (6.1)	60.8 (6.5)
Median	65.0	61.0	65.0	60.0
Sex				
Male	109 (54.5)	9888 (53.0)	74 (73.3)	68 (67.3)
Female	91 (45.5)	8780 (47.0)	27 (26.7)	33 (32.7)
Smoking status				
Current	98 (49.0)	3711 (19.9)	55 (54.5)	23 (22.8)
Former	102 (51.0)	14 957 (80.1)	46 (45.5)	78 (77.2)
Pack‐years (years)				
≤ 19	59 (29.5)	11 968 (64.1)	15 (14.9)	48 (47.5)
20–29	26 (13.0)	2246 (12.0)	17 (16.8)	21 (20.8)
30–39	31 (15.5)	1774 (9.5)	20 (19.8)	9 (8.9)
40–49	32 (16.0)	988 (5.3)	14 (13.9)	8 (7.9)
≥ 50	41 (20.5)	1200 (6.4)	32 (31.7)	14 (13.9)
Missing	11 (5.5)	492 (2.6)	3 (3.0)	1 (1.0)
Duration of smoking (years)				
< 15	15 (7.5)	8262 (44.3)	2 (2.0)	24 (23.8)
15–24	17 (8.5)	2819 (15.1)	6 (5.9)	22 (21.8)
25–34	21 (10.5)	2665 (14.3)	17 (16.8)	28 (27.7)
35–44	68 (34.0)	3356 (18.0)	43 (42.6)	19 (18.8)
≥ 45	79 (39.5)	1566 (8.4)	33 (32.7)	8 (7.9)
BMI				
Underweight (< 18.5 kg/m^2^)	4 (2.0)	179 (1.0)	2 (2.0)	0 (0)
Normal (18.5–24.9 kg/m^2^)	56 (28.0)	4933 (26.4)	31 (30.7)	18 (17.8)
Overweight (25.0–29.9 kg/m^2^)	85 (42.5)	8187 (43.9)	45 (44.6)	52 (51.5)
Obese (≥ 30 kg/m^2^)	52 (26.0)	5070 (27.2)	22 (21.8)	30 (29.7)
Missing	3 (1.5)	299 (1.6)	1 (1.0)	1 (1.0)
Follow‐up year (0–6) cases				
1	37 (18.5)	–	19 (18.8)	–
2	41 (20.5)	–	12 (11.9)	–
3	28 (14.0)	–	18 (17.8)	–
4	27 (13.5)	–	12 (11.9)	–
5	35 (17.5)	–	23 (22.8)	–
6	32 (16.0)	–	17 (16.8)	–

**Fig. 2:**
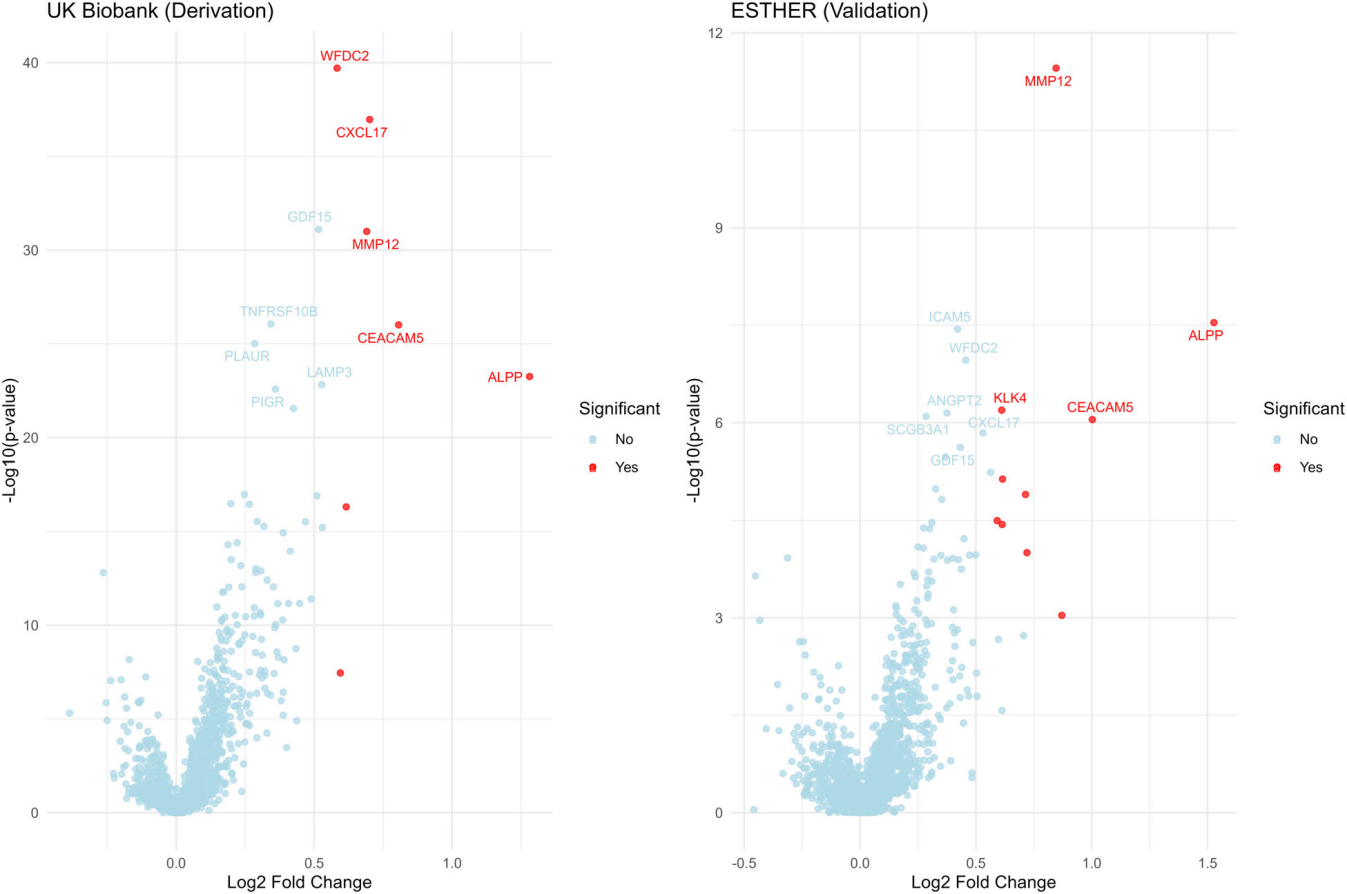
Volcano plots displaying differential protein expression between incident lung cancer cases and participants who did not develop lung cancer during 6 years of follow‐up among participants of UK Biobank (*n* LC = 200, *n* LC free = 18 668) and ESTHER (*n* LC = 101, *n* LC free = 101), based on NPX protein values. The *x*‐axis and the *y*‐axis show the log_2_ FC and the –log_10_ transformed *P*‐values from the Wilcoxon rank‐sum test. Points in red represent proteins significantly altered at an FDR threshold of < 0.05 and absolute log_2_ FC > 0.58 (1.5 FC) and those in blue represent only an FDR threshold of < 0.05. Labels indicate the top 10 proteins with the lowest FDR values. ALPP, alkaline phosphatase placental type; ANGPT2, angiopoietin‐2; CEACAM5, carcinoembryonic antigen‐related cell adhesion molecule 5; CXCL17, C‐X‐C motif chemokine 17; FC, fold change; FDR, false discovery rate; GDF15, growth differentiation factor 15; ICAM5, intercellular adhesion molecule 5; KLK4, kallikrein‐4; LAMP3, lysosome‐associated membrane glycoprotein 3; LC, lung cancer; MMP12, macrophage metalloelastase; *n*, number; NPX, normalized protein expression; PIGR, polymeric immunoglobulin receptor; PLAUR, urokinase plasminogen activator surface receptor; SCGB3A1, secretoglobin family 3A member 1; TNFRSF10B, tumor necrosis factor receptor superfamily member 10B; WFDC2, WAP four‐disulfide core domain protein 2.

The prediction performance of the 2025 proteins available in both datasets is presented in Table S1. After adjustment for multiple testing, the differences in protein concentrations between LC cases and participants that remained free of LC were statistically significant (*P*‐values < 0.05) for 551 proteins and 44 proteins, in the derivation and validation sets, respectively. AUCs ≥ 0.70 were observed for 11 and 9 proteins (highlighted in green in Table S1) in the derivation and validation sets, respectively. The volcano plots displaying the differential expressions of all 2 025 proteins between incident LC cases and participants who did not develop LC during 6 years of follow‐up among participants of the UK Biobank and ESTHER are shown in Fig. 2.

**Fig. 3:**
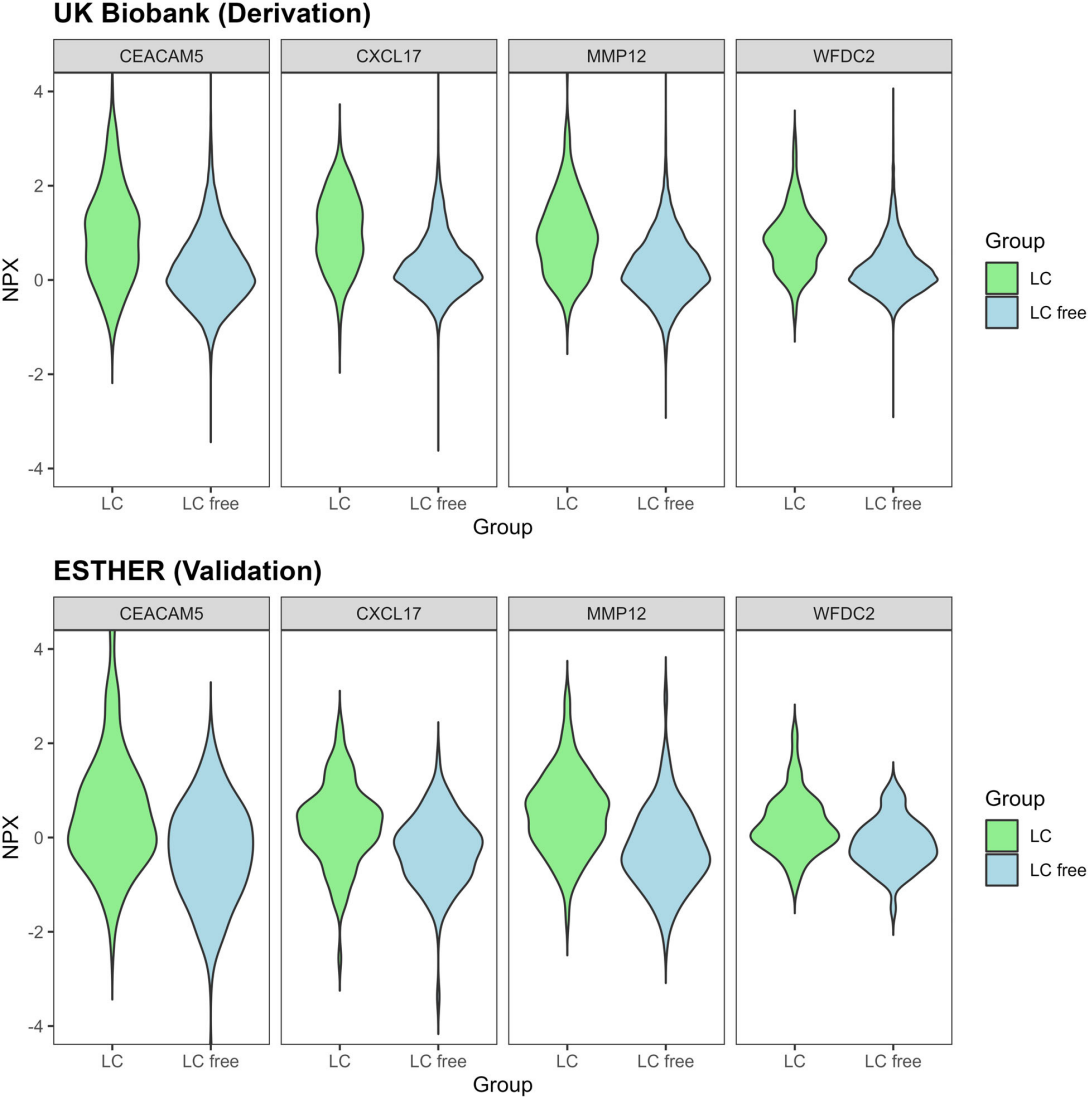
Violin plots showing the distribution of the four proteins selected in the protein marker model among incident lung cancer cases and participants who did not develop LC during 6 years of follow‐up among participants of UK Biobank (*n* LC = 200, *n* LC free = 18 668) and ESTHER (*n* LC = 101, *n* LC free = 101). CEACAM5, carcinoembryonic antigen‐related cell adhesion molecule 5; CXCL17, C‐X‐C motif chemokine 17; LC, lung cancer; MMP12, macrophage metalloelastase; *n*, number; NPX, normalized protein expression; WFDC2, WAP four‐disulfide core domain protein 2.

To identify a multi‐marker predictive signature distinguishing LC cases from LC‐free controls, we applied LASSO logistic regression on 2 025 protein biomarkers in the derivation set, with internal validation via bootstrap resampling. Although several biomarkers were differentially abundant in the derivation set, four proteins were selected by multivariate LASSO in at least 800 of 1 000 training sets and these four biomarkers were included in the final protein marker model. As shown in Table 2, the protein marker model predicted the incidence of LC in the derivation set with an AUC of 0.814 (95% CI, 0.785–0.843). The four proteins included in the protein marker model were carcinoembryonic antigen‐related cell adhesion molecule 5 (CEACAM5), C‐X‐C motif chemokine 17 (CXCL17), macrophage metalloelastase (MMP12), and WAP four‐disulfide core domain protein 2 (WFDC2). The estimates of β‐coefficients, odds ratios, and feature selection stability frequency of the four proteins selected in the final model in the derivation set are reported in the Table S2. In the validation set, an AUC of 0.814 (95% CI, 0.756–0.873) was observed for the protein marker model. As shown in Table S1, the AUCs for the four proteins CEACAM5, CXCL17, MMP12, and WFDC2 selected in the multi‐marker signature individually were 0.72, 0.76, 0.74, and 0.77 in the derivation set and 0.70, 0.70, 0.78, and 0.72 in the validation set, respectively. The violin plots displaying the distribution of the four proteins selected in the protein marker model among incident LC cases and participants who did not develop LC during 6 years of follow‐up among participants of the UK Biobank and ESTHER are shown in Fig. 3.

**Table 2:** Potential of predictors for identifying incident lung cancer cases among ever‐smoking participants of the UK Biobank and ESTHER. 95% CI, 95% confidence interval; AUC, area under the receiver operating curve; LC, lung cancer; *n*, number; PLCO_m2012_, Prostate, Lung, Colorectal, and Ovarian Cancer Screening Trial Model 2012.

Predictor	UK Biobank (derivation) (*n* LC = 200, *n* LC free = 18 668)		ESTHER (validation) (*n* LC = 101, *n* LC free = 101)	
	AUC (95% CI)	Improvement	AUC (95% CI)	Improvement
Protein marker model	0.814 (0.785–0.843) 0.813^a^	–	0.814 (0.756–0.873) 0.813^a^	–
PLCO_m2012_	0.758 (0.723–0.793)	–	0.775 (0.710–0.841)	–
PLCO_m2012_ + protein marker model	0.814 (0.785–0.843) 0.813^a^	0.056^b^	0.832 (0.776–0.888) 0.831^a^	0.057^b^

a Denotes the bootstrap (overoptimism corrected) estimates of the area under the receiver operating curve.

b Denotes that the *P*‐value presented from the DeLong test is < 0.05 for assessing the differences in area under the receiver operating curves between the PLCO_m2012_ only and the combined PLCO_m2012_ + protein marker model.

**Fig. 4:**
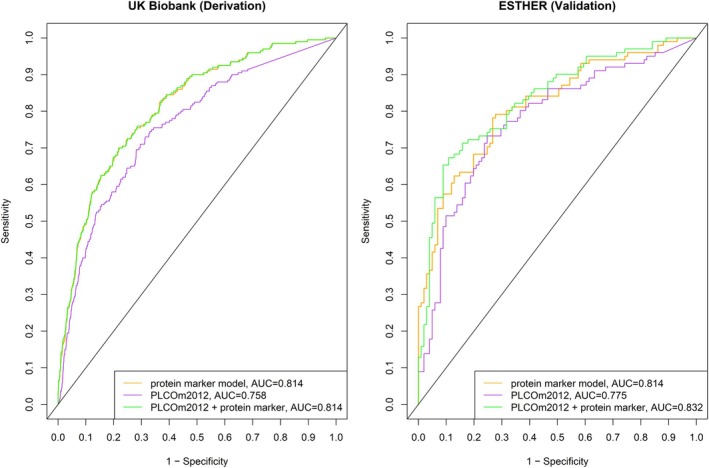
Receiver operating characteristic curves of protein marker model and PLCO_m2012_, individually and in combination, for predicting lung cancer incidence in UK Biobank (*n* LC = 200, *n* LC free = 18 668) and ESTHER (*n* LC = 101, *n* LC free = 101). AUC, area under the receiver operating curve; LC, lung cancer; *n*, number; PLCO_m2012_, Prostate, Lung, Colorectal, and Ovarian Cancer Screening Trial Model 2012.

The predictive performance of the protein marker model and PLCO_m2012_ model, alone and in combination, for the derivation and validation sets is presented in Table 2 and Fig. 4. Individually, the protein marker model outperformed the PLCO_m2012_ model. The overall AUCs for the PLCO_m2012_ model in the derivation and validation sets were 0.758 (95% CI, 0.723–0.793) and 0.775 (95% CI, 0.710–0.841). When adding the protein marker model to the PLCO_m2012_ model, the AUC was estimated at 0.814 (95% CI, 0.785–0.843) in the derivation and at 0.832 (95% CI, 0.776–0.888) in the validation set. As shown in Table 3, the odds ratios per increase in the protein marker model by 1 standard deviation were 2.62 (95% CI, 2.36–2.92) for the derivation set and 4.79 (95% CI, 2.97–7.71) for the validation set, respectively.

**Table 3:** Performance of the derived protein marker model to predict incident lung cancer cases. 95% CI, 95% confidence interval; LC, lung cancer; *n*, number.

Protein marker model^a^	UK Biobank (derivation) (*n* LC = 200, *n* LC free = 18 668)				ESTHER (validation) (*n* LC = 101, *n* LC free = 101)			
	LC free (*n*)	LC (*n*)	Odds ratios (95% CI)	*P*‐value	LC free (*n*)	LC (*n*)	Odds ratios (95% CI)	*P*‐value
Quartile 1	4667 (25.0)	4 (2.0)	–	< 0.001	26 (26.0)	7 (6.9)	–	< 0.001
Quartile 2	4667 (25.0)	16 (80.0)	4.00 (1.34–11.97)		25 (25.0)	8 (7.9)	1.19 (0.38–3.77)	
Quartile 3	4667 (25.0)	33 (19.0)	8.25 (2.92–23.30)		24 (24.0)	14 (13.9)	2.17 (0.75–6.28)	
Quartile 4	4667 (25.0)	147 (71.0)	3.68 (1.16–89.28)		26 (26.0)	72 (71.3)	10.29 (4.00–26.53)	
OR per SD	18 668	200	2.62 (2.36–2.92)	< 0.001	101	101	4.79 (2.97–7.71)	< 0.001

a Categorized according to quartiles among participants free of LC.

The sensitivities of the protein marker model, individually and in combination with the PLCO_m2012_ model, the four definitions of heavy smoking criteria used by different LDCT trials and the USPSTF 21 guideline, for predicting incident LC cases during a 6‐year follow‐up are provided in Table 4. Using the trial criteria and USPSTF guidelines in the derivation set, between 51.5% (NLST) and 62.5% (USPSTF 21) of LC cases would have been correctly identified as eligible for screening. At cutoffs yielding the same number of participants selected for LDCT screening as the respective trial criterion and guideline, the sensitivities (proportions of correctly predicted LC cases) would have ranged from 55.5% to 64.5% for the PLCO_m2012_ model and from 65.0% to 73.5% for the protein marker model, respectively (Table 4). In the validation set, between 59.4% (NLST) and 73.3% (USPSTF 21) of LC cases would have been correctly identified as eligible for screening. The sensitivities ranged from 65.3% to 77.2% and from 66.3% to 79.2% for the PLCO_m2012_ model and the protein marker model, respectively. The increase in sensitivity compared to the trial criterion was highest for the DEPISCAN criterion (+16 and +12 percent units, *P* < 0.05 in the derivation and validation set, respectively). In the derivation and validation sets, the combination of the PLCO_m2012_ model with the protein marker model did not further improve the sensitivities as compared to the protein marker model alone.

**Table 4:** Comparison of selection criteria used in LDCT Trials with that of protein marker model, PLCO_m2012_ model, individually and in combination^a^, for predicting lung cancer incidence during 17 years of follow‐up among ever‐smoker ESTHER participants. cig, cigarettes; DANTE, Detection and Screening of Early Lung cancer with Novel Imaging Technology; DLCST, Danish Lung Cancer Screening Trial; ITALUNG, Italian Lung Cancer Computed Tomography Screening Trial; LC, lung cancer; LDCT, low‐dose computed tomography; LUSI, German Lung Cancer Screening Intervention trial; MILD, Multicentric Italian Lung Detection Trial; NELSON, *Nederlands‐Leuvens Longkanker Screenings Onderzoek* trial; NLST, United States National Lung Screening Trial; *n*, number; PLCO_m2012_, Prostate, Lung, Colorectal, and Ovarian Cancer Screening Trial Model 2012; USA, United States of America; USPSTF, US Preventive Services Task Force.

Trial, country	NLST, USA [2]	MILD, Italy [21], DANTE, Italy [22], ITALUNG, Italy [3], DLCST, Denmark [23]	NELSON, Netherlands/Belgium [25], LUSI, Germany [5]	DEPISCAN, France [26]	USPSTF 2020 [24]
Criterion	≥ 30 pack‐years	≥ 20 pack‐years	≥ 15 cig per day for ≥25 years or ≥ 10 cig per day for ≥ 30 years	≥ 15 cig per day for ≥ 20 years	≥ 20 pack‐years
Restriction for former smokers	Quit ≤ 15 years ago	Quit ≤ 10 years ago	Quit ≤ 10 years ago	Quit ≤ 15 years ago	Quit ≤ 15 years ago

	Sensitivity, *n* (%)	Sensitivity, *n* (%)	Sensitivity, *n* (%)	Sensitivity, *n* (%)	
UK Biobank (Derivation)					
Trial criterion	103 (51.5%)	113 (56.5%)	121 (60.5%)	114 (57.0%)	125 (62.5%)
PLCO_m2012_	111 (55.5%)	119 (59.5%)	124 (62.0%)	129 (64.5%)^b^	129 (64.5%)
Protein marker model	130 (65.0%)^b^	140 (70.0%)^b^	144 (72.0%)^b^	146 (73.0%)^b^	147 (73.5%)^b^
PLCO_m2012_ + protein marker model	129 (64.5%)^b^	140 (70.0%)^b^	145 (72.5%)^b^	146 (73.0%)^b^	147 (73.5%)^b^
ESTHER (Validation)					
Trial criterion	60 (59.4%)	66 (65.3%)	64 (63.4%)	68 (67.3%)	74 (73.3%)
PLCO_m2012_	66 (65.3%)	72 (71.3%)	72 (71.3%)	78 (77.2%)	78 (77.2%)
Protein marker model	67 (66.3%)	73 (72.3%)	73 (72.3%)	80 (79.2%)^b^	80 (79.2%)
PLCO_m2012_ + protein marker model	67 (66.3%)	73 (72.3%)	73 (72.3%)	80 (79.2%)^b^	80 (79.2%)

a To ensure comparability, cutoffs of protein marker model, PLCO_m2012_ and their combinations were adjusted in such a way that the same number of controls remaining free of LC were classified as non‐eligible as with the respective trial criteria.

b Denotes that the *P*‐value from the McNemar test is < 0.05 for the difference in sensitivity compared to the respective LDCT trial criterion.

## 4 Discussion

In this study, we developed a novel protein‐based risk prediction tool which outperformed the PLCO_m2012_ model in both the internal and external validation cohorts of target use population for screening. A protein marker model comprising four markers was derived based on the participants from the UK Biobank study, and subsequently, the performance was evaluated in an independent validation set comprising participants of the ESTHER study. The protein marker model outperformed the PLCO_m2012_ model in both the derivation and validation sets. At cutoffs yielding the same proportion and number of participants selected for LDCT screening as the respective trial criterion, the protein marker model predicted occurrence of LC within 6 years with up to 16 and 12 percent units higher sensitivity compared to the trial criteria in the derivation and validation set, respectively.

Following the demonstration of reduction of LC mortality by LDCT screening in large‐scale randomized trials, efforts to implement LC screening programs are ongoing in many countries. At the same time, major efforts are made to find ways to optimize the effectiveness and cost‐effectiveness of the programs by enhanced selection of participants most likely to benefit from screening. Apart from risk factor‐based risk prediction models, blood‐based biomarkers could be potentially promising, easy‐to‐implement tools in this context [29]. In recent years, a wide array of blood‐based biomarkers have been proposed for LC risk prediction [13, 14, 30, 31]. However, only a few of these biomarkers have been independently validated and none are widely used in screening [32].

In this study, a four‐protein biomarker signature was derived and found to outperform risk factor‐based risk prediction models in both internal and external validation. The four proteins included in the LASSO‐derived protein marker model were CEACAM5, CXCL17, MMP12, and WFDC2. Expression of CEACAM5, a cell surface glycoprotein, has been identified in several cancers, including lung cancer. MMP12, which is a metalloproteinase secreted by macrophages, has been found to be aberrantly expressed in various tumors. Protein biomarkers like CEACAM5 and MMP12 have been previously included in protein biomarker risk prediction tools for LC risk prediction [14, 16]. CXCL17 is a member of the chemokine family and its expression has been observed in patients with both nonmalignant and malignant diseases [33]. In the current study, these four LC‐associated proteins performed equally well, individually and as a model, in both the UK Biobank and ESTHER for predicting LC among ever‐smoking participants.

The primary strength of this study is the two‐stage design, utilizing prediagnostic blood samples from large population‐based cohorts. This study performed comprehensive protein discovery using blood samples from the UK Biobank study and then externally validated findings in participants from ESTEHR. Using next‐generation high‐throughput proteomics, in the form of Olink® Explore 3072 and Olink® Explore HT arrays, comprehensive protein expression patterns were screened to identify markers that could enable prediction of lung cancer up to 6 years before diagnosis. Advanced statistical machine learning algorithms were employed to simultaneously evaluate possible combinations and comparisons of the protein markers. Our analysis included LC cases occurring during up to 6 years of follow‐up which makes the results closely comparable to the PLCO_m2012_ which was designed to predict lung cancer incidence within periods of 6 years. From a clinical application standpoint, the protein marker model comprises only four proteins, making it easily applicable and efficient.

Although the current study provided evidence of the potential of using protein biomarkers in lung cancer risk assessment to define screening eligibility, further validation of this risk prediction model using prediagnostic samples in a larger population is needed before the use of this risk prediction tool in practice. Validation in a further, larger cohort should also include more detailed analyses for specific subgroups, for example, defined by age and sex. In previous biomarker verification studies, proximity extension assays have been found to give higher throughput, good sensitivity, and easier workflows as compared to other protein detection methods like mass‐spectrometry or antibody‐based arrays [34, 35]; nevertheless, replication of our findings using other quantification techniques should be aimed for. Furthermore, validation research should also include absolute quantification of the protein biomarker concentrations, for example by ELISA, rather than the relative quantification used in our study. Finally, thorough cost‐effective and benefit–harm assessments in large population cohorts will be crucial to best define the role of use of the biomarkers in risk‐adapted LC screening.

## 5 Conclusion

A protein marker model was developed to identify future lung cancer cases and its prediction potential was externally validated in an independent cohort. Using blood samples from population‐based cohorts, the protein marker model showed promising risk discriminative performance beyond the established PLCO_m2012_ model. Further research is required to define a potential role of using the blood protein‐based panel to enhance the effectiveness and cost‐effectiveness of LC screening.

## Conflict of interest

The authors declare no conflict of interest.

## Author contributions

MB—conceptualization; data curation; formal analysis; investigation; methodology; project administration; writing – original draft and writing – review and editing. HB—conceptualization; funding acquisition; project administration; resources; software; supervision. CF—project administration and writing – review and editing. BS, BH, and HB—responsible for the field work and work‐up of data of the ESTHER study and writing – review and editing. All authors critically reviewed the manuscript for important intellectual content and approved the final version for submission.

## Supporting information


**Fig. S1.** Preprocessing of the protein biomarkers in UK Biobank and ESTHER.

**Table S1.** Individual predictive performance of each of the 2025 protein biomarkers for predicting LC incidence within 6 years from blood samples in the derivation and validation sets (*P*‐values are from the Wilcoxon rank‐sum test).

**Table S2.** The estimates of β‐coefficients, odds ratios, and feature selection stability frequency of the proteins included in the final model.

## Data Availability

Due to restrictions of informed consent, the ESTHER study data cannot be made publicly available. However, use of the ESTHER data for collaboration projects has been and will remain the approach for data sharing. The UK Biobank data used for analysis are available for further research upon application to the data owners (https://www.ukbiobank.ac.uk/).
