# Supplementary material for: Next‐generation proteomics improves lung cancer risk prediction
Source: Mol Oncol. 2025 Nov 20;20(4):995–1007. doi: 10.1002/1878-0261.70166 (PMC13060633; doi:10.1002/1878-0261.70166)
Supplement: Supplementary file 1 — Fig. S1. Preprocessing of the protein biomarkers in UK Biobank and ESTHER. [file MOL2-20-995-s003.pptx]

## Slide 1
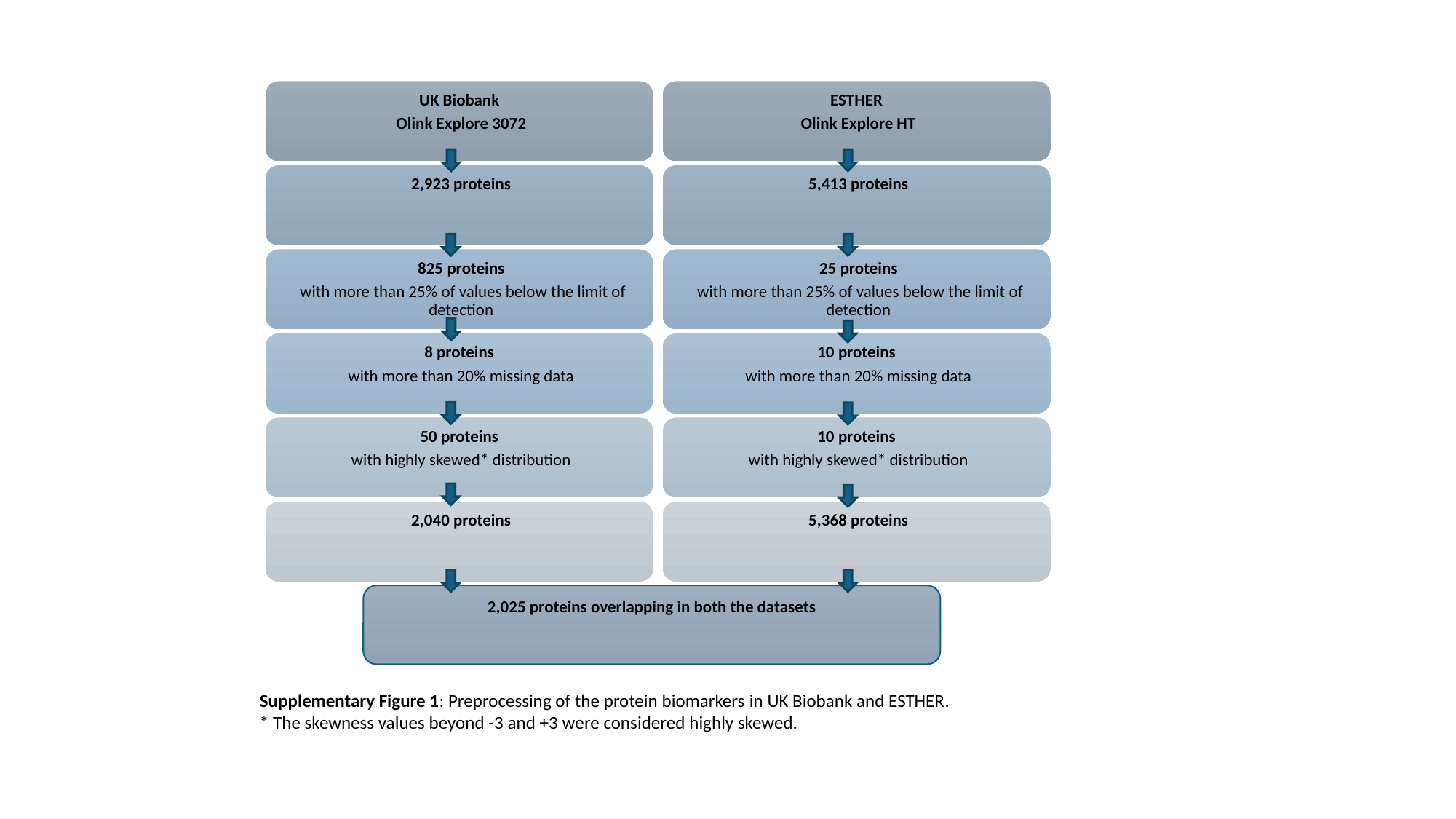

2,025 proteins overlapping in both the datasets
Supplementary Figure 1: Preprocessing of the protein biomarkers in UK Biobank and ESTHER.
* The skewness values beyond -3 and +3 were considered highly skewed.
