# Supplementary material for: Next‐generation proteomics improves lung cancer risk prediction
Source: Mol Oncol. 2025 Nov 20;20(4):995–1007. doi: 10.1002/1878-0261.70166 (PMC13060633; doi:10.1002/1878-0261.70166)
Supplement: Supplementary file 3 — Table S2. The estimates of β‐coefficients, odds ratios, and feature selection stability frequency of the proteins included in the final model. [file MOL2-20-995-s001.docx]

**Supplementary Table 2**: The estimates of β-coefficients, odds ratios and feature selection stability frequency of the proteins included in the final model

| **Variable** | **β coefficient** | **OR (95% CI)** | **p value** | **Selection Frequency** |
| --- | --- | --- | --- | --- |
| (Intercept) | -5.12 | 0.01 (0.00-0.01) | <0.00005 | - |
| CXCL17 | 0.27 | 1.31 (1.13-1.51) | <0.00005 | 100% |
| WFDC2 | 0.23 | 1.26 (1.09-1.45) | <0.00005 | 99.8% |
| CEACAM5 | 0.40 | 1.50 (1.33-1.68) | <0.00005 | 100% |
| MMP12 | 0.41 | 1.50 (1.30-1.73) | <0.00005 | 99.9% |

**Abbreviations**: **CEACAM5**- carcinoembryonic antigen-related cell adhesion molecule 5; **CXCL17**- C-X-C motif chemokine 17; **MMP12**- macrophage metalloelastase**; OR-** Odds ratios; **WFDC2**- WAP four-disulfide core domain protein; **95% CI**- 95 % confidence interval.
